# Efficacy of Trimethoprim–Sulfamethoxazole in Combination with an Echinocandin as a First-Line Treatment Option for Pneumocystis Pneumonia: A Systematic Review and Meta-Analysis

**DOI:** 10.3390/antibiotics11060719

**Published:** 2022-05-26

**Authors:** Hideo Kato, Mao Hagihara, Nobuhiro Asai, Takumi Umemura, Yuichi Shibata, Jun Hirai, Yuka Yamagishi, Takuya Iwamoto, Hiroshige Mikamo

**Affiliations:** 1 Department of Clinical Infectious Diseases, Aichi Medical University, Nagakute 480-1195, Japan; katou.hideo.233@mail.aichi-med-u.ac.jp (H.K.); hagimao@aichi-med-u.ac.jp (M.H.); nobuhiro0204@gmail.com (N.A.); umemuratakumi@gmail.com (T.U.); shibata.yuuichi.414@mail.aichi-med-u.ac.jp (Y.S.); hiraichimed@gmail.com (J.H.); y.yamagishi@mac.com (Y.Y.); 2 Department of Pharmacy, Mie University Hospital, Tsu 514-8507, Japan; taku-iwa@med.mie-u.ac.jp; 3 Department of Clinical Pharmaceutics, Division of Clinical Medical Science, Mie University Graduate School of Medicine, Tsu 514-8507, Japan; 4 Department of Molecular Epidemiology and Biomedical Sciences, Aichi Medical University Hospital, Nagakute 480-1195, Japan

**Keywords:** meta-analysis, pneumocystis pneumonia, trimethoprim–sulfamethoxazole, echinocandin, combination therapy

## Abstract

Although combination therapy using trimethoprim–sulfamethoxazole (TMP–SMX) plus echinocandins has been reported to reduce the mortality of patients with pneumocystis pneumonia (PCP), it remains unclear whether it is more effective than TMP–SMX monotherapy, the current first-line treatment for this disease. Hence, we performed a systematic review and meta-analysis to compare the efficacies of these treatment options for PCP. The Scopus, EMBASE, PubMed, CINAHL, and Ichushi databases were searched for studies (up to January 2022) reporting the mortality and positive response rates (fewer clinical symptoms, improved partial pressure of arterial oxygen, and resolution of pneumonitis on chest imaging) of PCP patients receiving monotherapy or combination therapy. Four studies met the inclusion criteria. All four presented mortality data and one had positive response rates. Compared with the monotherapy, the combination therapy resulted in significantly lower mortality and higher positive response rates (mortality: odds ratio (OR) 2.20, 95% confidence interval (CI) 1.46–3.31; positive response rate: OR 2.13, 95%CI 1.41–3.23), suggesting it to be an effective and promising first-line therapy for PCP. However, further safety evaluations are needed to establish this as a fact.

## 1. Introduction

Pneumocystis pneumonia (PCP), a lung disease caused by infection by the opportunistic fungus *Pneumocystis jirovecii*, occurs mainly in immunocompromised patients, including individuals infected with the human immunodeficiency virus (HIV) and those receiving immune suppression treatments [1,2]. The mortality rates associated with PCP remain as high as 20–48% among HIV-positive patients and 20–60% among non-HIV-infected patients [3,4]. Therefore, the early selection of appropriate antibiotic agents is particularly important for combating this disease.

The current use of trimethoprim–sulfamethoxazole (TMP–SMX) as the first-line drugs for the treatment of PCP has been unchanged for many years [5]. However, serious adverse events associated with TMP–SMX monotherapy and resistance to the drugs have been reported. Approximately 25% of PCP patients are unable to complete the full course of TMP–SMX monotherapy owing to treatment failure or various side effects, such as bone marrow suppression, renal damage, and gastrointestinal upset [6]. Moreover, the widespread and long-term prophylaxis of this disease with TMP–SMX has led to the development of sulfa drug-resistant *P. jirovecii* [7].

Alternative agents, including echinocandins, have been investigated for their therapeutic potential in treating PCP. Previous studies conducted with a limited number of patients have reported divergent findings on echinocandin monotherapy for PCP [8], whereas combination regimens of TMP–SMX plus echinocandins have improved the prognosis and reduced the mortality associated with this disease [9,10]. However, it remains unclear whether TMP–SMX + echinocandin combination therapy is more effective than TMP–SMX monotherapy for the treatment of PCP.

To date, there is only one case-control study with available clinical data on the effects of TMP–SMX in combination with echinocandins on PCP [11]. Recently, several retrospective cohort studies comparing TMP–SMX monotherapy with the combination therapy for PCP have been published [12,13,14]. Although each study showed a tendency of the combination therapy to improve the PCP mortality rate, they did not provide obvious evidence for its use as the preferred treatment strategy. Hence, we performed a systematic review and meta-analysis to evaluate the feasibility of using TMP–SMX in combination with an echinocandin as a first-line treatment option for PCP.

## 2. Results

### 2.1. Systematic Review

**Figure 1:**
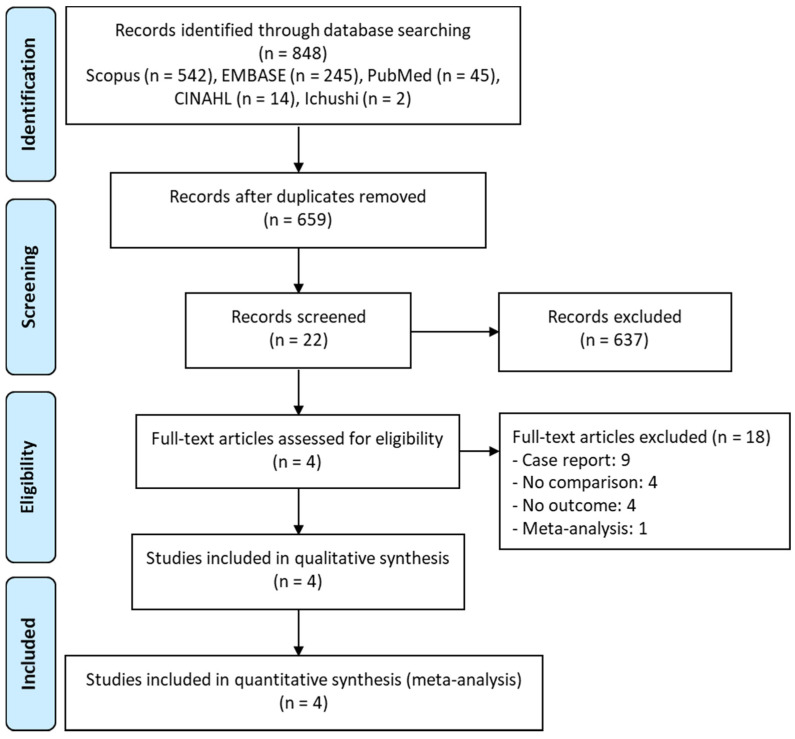
PRISMA flow diagram of the selection of eligible studies.

Five major scholarly databases were searched for articles (up to January 2022) related to TMP–SMX monotherapy and TMP–SMX + echinocandin combination therapy for PCP. Of the 848 potentially relevant articles that were retrieved, the titles and abstracts of 659 papers were screened after the removal of duplicates. Subsequently, the full-text review of 22 articles was performed. Of these, 18 articles were further excluded on the basis of the exclusion criteria listed in Figure 1, leaving four studies that met eligibility for the meta-analysis [11,12,13,14].

The characteristics of these four studies, all of which were conducted at single centers, are summarized in Table 1. One was a case-control study conducted in Taiwan [11], and the other three were cohort studies undertaken in China [12,13,14]. The participants reported by Lu et al. [11] were heart transplant recipients, whereas those in the cohort studied by Jin et al. [12] were non-HIV-infected patients. By contrast, the participants reported by Wang et al. [13] and Tian et al. [14] were all HIV-infected patients. All the patients in all four studies were adults [11,12,13,14]. In total, 301 received TMP–SMX monotherapy and 235 received TMP–SMX + echinocandin combination therapy. The following TMP–SMX dosage regimens were used in the respective studies: TMP 5.5–20 mg/kg/day [11]; dosage recommended by international guidelines [12]; TMP 80 mg/day and SMX 400 mg/day [13]; and TMP 15–20 mg/kg/day and SMX 75–100 mg/kg/day [14]. The echinocandins administered were caspofungin (50 mg/day, 70 mg on day 1) or anidulafungin (100 mg/day, 200 mg on day 1) in the study by Lu et al. [11] and caspofungin (50 mg/day, 70 mg on day 1) in the other three studies [12,13,14]. The risk-of-bias assessment scores based on the Newcastle–Ottawa Quality Assessment Scale are presented in Table 1. The median score was 7, and the range was 5–8.

### 2.2. Meta-Analysis

#### 2.2.1. Mortality

**Figure 2:**
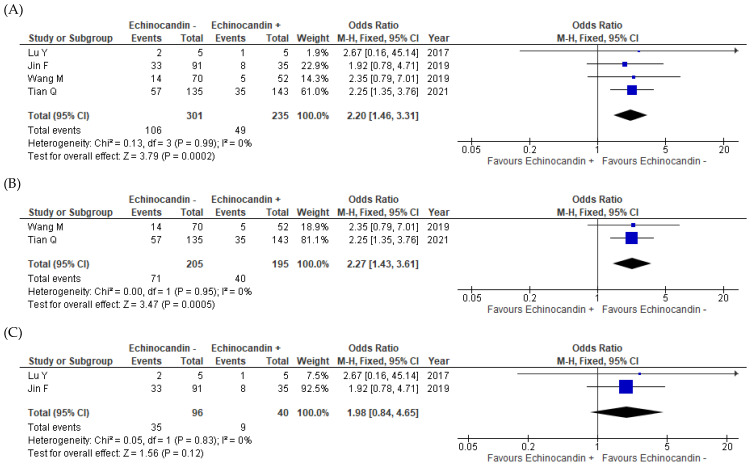
Forest plot of odds ratios for the comparisons of mortality outcomes between patients with pneumocystis pneumonia who received TMP–SMX + echinocandin combination therapy and those who received TMP–SMX monotherapy. (**A**) Overall results for all patients with pneumocystis pneumonia; (**B**) HIV-infected patients with pneumocystis pneumonia; (**C**) Non-HIV-infected patients with pneumocystis pneumonia. echinocandin −, TMP–SMX monotherapy; echinocandin +, TMP–SMX + echinocandin combination therapy; M–H, Mantel–Haenszel; CI, confidence interval; HIV, human immunodeficiency virus; OR, odds ratio; SMX, sulfamethoxazole; TMP, trimethoprim.

All four studies reported mortality data [11,12,13,14]. The rates were 35.2% (106/301) for the monotherapy groups and 20.9% (49/235) for the combination therapy groups of all studies combined. As indicated by the meta-analysis, the combination therapy appeared to have resulted in significantly improved mortality outcomes relative to the effects of the monotherapy (odds ratio (OR) = 2.20, 95% confidence interval (CI) = 1.46–3.31, *I*^2^ = 0%; Figure 2A). When stratified by HIV infection status, this positive effect of the combination therapy on mortality outcomes was true for the HIV-infected patients (OR = 2.27, 95% CI = 1.43–3.61, *I*^2^ = 0%; Figure 2B) [13,14] but not for the non-HIV-infected participants (OR = 1.98, 95% CI = 0.84−4.65, *I*^2^ = 0%; Figure 2C) [11,12]. However, the combination therapy significantly improved the mortality outcomes for non-HIV-infected patients with severe PCP (OR = 5.07, 95% CI = 1.40–18.37) [12].

#### 2.2.2. Positive Response Rates

**Figure 3:**
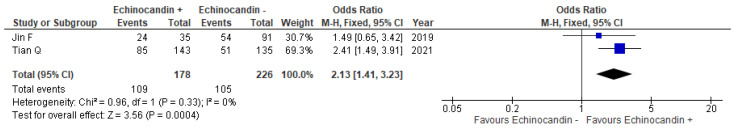
Forest plot of odds ratios for the comparisons of positive response rates between patients with pneumocystis pneumonia who received TMP–SMX + echinocandin combination therapy and those who received TMP–SMX monotherapy. echinocandin −, TMP–SMX monotherapy; echinocandin +, TMP–SMX + echinocandin combination therapy; M–H, Mantel–Haenszel; CI, confidence interval; OR, odds ratio; SMX, sulfamethoxazole; TMP, trimethoprim.

Data regarding positive response rates were reported in two studies. The overall rates were 46.5% (105/226) for the monotherapy group and 61.2% (109/178) for the combination therapy group [12,14]. Compared with the monotherapy, the combination therapy resulted in significantly better positive response rates (OR = 2.13, 95% CI = 1.41–3.23, *I*^2^ = 0%; Figure 3).

## 3. Discussion

The present study demonstrated that TMP–SMX in combination with an echinocandin was associated with improved mortality outcomes for patients with PCP compared with the results from TMP–SMX monotherapy. In the group of HIV-infected patients with PCP, the combination therapy resulted in significantly better mortality outcomes and positive response rates than those obtained with the monotherapy. In the groups of non-HIV-infected patients, the combination therapy tended to provide better mortality outcomes than those from the monotherapy, and it significantly reduced the mortality rate in the patients with severe PCP.

Although originally classified as a protozoan, *P. jirovecii* is now considered to have fungal characteristics. Drugs typically used for the treatment of fungal infections target ergosterol and beta-(1,3)-d-glucan in the cell membrane of the fungus. However, because the sterol in the cell membrane of *P. jirovecii* is cholesterol and not fungal ergosterol [15,16], azoles and polyenes are considered ineffective against this pathogen. In fact, to the best of our knowledge, there are no known studies in which these fungal agents were used for the treatment of PCP. By contrast, echinocandins that target beta-(1,3)-d-glucan have attracted increased attention for the treatment of PCP. The feasibility of combining TMP–SMX with echinocandin for PCP therapy has been demonstrated in animal models of the disease and in vitro experiments [17,18,19], reinforcing our findings that the combination therapy is a promising regimen.

The principal behind the therapeutic effect of TMP-SMX + echinocandin on PCP has been demonstrated using animal models of the disease and in vitro experiments [20,21,22]. Although echinocandins can block the formation of *P. jirovecii* cysts by interfering with the synthesis of beta-(1,3)-d-glucan, they have low efficacy against trophozoite forms [23]. These findings indicate that echinocandins can reduce pathogen reservoirs. By contrast, TMP–SMX inhibits trophozoites by inhibiting their metabolism of folate [20]. Moreover, the echinocandins act quickly, whereas the onset of the therapeutic effects of TMP–SMX occurs only after 5–8 days [24]. The present study indicated that the combination therapy significantly improved the mortality outcomes of overall patients with PCP relative to the results obtained with TMP–SMX monotherapy. Therefore, in the treatment of patients with PCP, the combination therapy is expected to execute its synergistic effects earlier by inhibiting the entire life cycle of *P. jirovecii*.

A previous study has reported that HIV-infected patients carried a significantly greater burden of *P. jirovecii* in lung lavages compared with non-HIV-infected patients [25]. Moreover, the trophic forms are generally more abundant during pneumocystis pneumonia (PCP), but there are no data to show the percentage of trophic and cystic forms during PCP [26]. Therefore, the combination therapy may not be so effective, since the number of cystic forms which echinocandins target is relatively low in non-HIV-infected patients.

The incidence of adverse events from echinocandins is very low [27]. A review of clinical trials showed that less than 3% of patients experienced severe adverse events or discontinued treatment as a result of echinocandin-related adverse events [28]. In fact, there were no serious events caused by TMP–SMX + echinocandin combination therapy, and no patients discontinued the combination therapy because of clinical or laboratory adverse events [12]. Therefore, the combination therapy may not have any higher risk than the monotherapy. However, because only one study reported on the safety of the combination therapy, further validation studies are needed to verify its risk–benefit profile [12].

To the best of our knowledge, this is the first meta-analysis of the efficacy of TMP–SMX + echinocandin combination therapy as a first-line treatment for PCP patients. However, our meta-analysis has several limitations. First, the number of studies included was relatively small, despite that subgroup analyses on patient background and disease severity were performed. Moreover, because only single-center, retrospective studies were included, three of which were conducted in China, the likelihoods of reporting and selection biases may have been increased, although there was no heterogeneity (*I*^2^ = 0%) in our results. Second, results of drug resistance in the PCP patients were not reported. In fact, the culturing of *P. jirovecii* has not yet been established.

## 4. Materials and Methods

### 4.1. Study Design and Data Sources

This study was conducted according to PRISMA guidelines, except for the protocol registration for reporting systematic reviews and meta-analyses (File S1) [29,30]. The following PICO criteria were used for selecting relevant studies: population (P), patients with PCP, intervention (I), patients receiving TMP–SMX monotherapy, comparison (C), patients receiving TMP–SMX + echinocandin combination therapy, outcome (O), mortality, and positive response rate. All studies up to 7 January 2022 were identified through a systematic review of publications on the Scopus, EMBASE, PubMed, CINAHL, and Ichushi databases. The search keywords used were “*Pneumocystis jirovecii*”, “anidulafungin”, “caspofungin”, “micafungin”, and “echinocandin”. Language was restricted to English and Japanese. Additional searches were conducted by analyzing the references from retrieved papers and reviews to minimize the chance of omissions.

### 4.2. Study Selection

To avoid bias, the literature search based on titles and abstracts was performed independently by two of the authors, and then the full-text articles were reviewed to extract appropriate studies for this meta-analysis. A third author (H.M.) resolved any disagreements by discussion. Studies that met the following criteria were extracted: (i) randomized controlled trial, retrospective observational, or cohort studies; (ii) having patients diagnosed with pneumocystis pneumonia; and (iii) having patients receiving TMP–SMX monotherapy or TMP–SMX + echinocandin combination therapy. No restriction was placed on the regimen and duration of antibiotic treatment. Non-clinical studies and review publications were excluded, as were case reports with sample sizes of less than 5 patients. Exclusion of studies with relatively small sample sizes was intended to minimize selection and reporting bias. Subgroup analyses were performed with the following populations: non-HIV-infected patients, non-HIV-infected patients with severe PCP, and HIV-infected patients. Severe PCP was defined as a partial pressure of arterial oxygen (PaO_2_)/fraction of inspired oxygen (FiO_2_) of less than 60 mmHg or an alveolar–arterial oxygen difference (D_(A − a)_O_2_) of greater than 45 mmHg, and a beta-d-glucan concentration of over 800 pg/mL.

### 4.3. Data Extraction and Risk-of-Bias Assessment

The following data were extracted independently from each study: study design, setting, and period; country of study; drug regimen; number of eligible patients; ages of patients; population; and clinical outcomes. The primary outcome was all-cause mortality. The secondary outcome was a positive response rate, which was defined as fewer clinical symptoms, an improved PaO_2_ value, and resolution of the pneumonitis on chest imaging after treatment. The Newcastle–Ottawa Quality Assessment tool was used to evaluate the risk-of-bias of retrospective studies [31]. The tool consists of three domains: selection, comparability, and exposure.

### 4.4. Statistical Analysis

Data analysis was performed using Review Manager software (RevMan, version 5.4; Cochrane Collaboration, Oxford, UK) according to a previous study [32]. The degree and proportion of statistical heterogeneity were evaluated using the chi-squared test and the *I*^2^ measure, respectively. The heterogeneity was defined as being significant when the p value was less than 0.1 or the *I*^2^ value was greater than 50%. The random-effects model was applied for heterogeneous data and the fixed-effects model for homogeneous data. The pooled OR and 95% CIs were calculated.

## 5. Conclusions

In conclusion, our meta-analysis showed that TMP–SMX in combination with an echinocandin significantly reduced the mortality rates of HIV-infected patients with PCP and non-HIV-infected patients with severe PCP. Our findings indicate that TMP–SMX + echinocandin combination therapy is an effective and promising first-line treatment option for severe PCP, especially if initiated in the early stage of the disease.

## Figures and Tables

**Table 1:** Characteristics of the studies included in the meta-analysis.

Study	Study Design	Setting	Period	Country of Study	Drug Regimen TMP-SMX	Echinocandin	No. of Eligible Patients (Echinocandin − vs. Echinocandin +)	Age (Year) Median (Minimum–Maximum)	Population	Clinical Outcome	Risk-of-Bias Score
Lu. Y., 2017 [11]	Retrospective case control study	Single-center	July 1988 to December 2015	Taiwan	TMP 5.5–20 mg/kg/day	Caspofungin 50 mg/day, 70 mg on day 1; anidulafungin 100 mg/day, 200 mg on day 1	5 vs. 5	Overall 56 (34–76)	Heart transplant recipients	Mortality	5
Jin, F., 2019 [12]	Retrospective cohort study	Single-center	January 2012 to June 2018	China	Dosage recommended by international guidelines	Caspofungin 50 mg/day, 70 mg on day 1	91 vs. 35	Overall 57	Patients without HIV infection	Mortality; Positive response rate	7
Wang, M., 2019 [13]	Retrospective cohort study	Single-center	January 2013 to June 2018	China	TMP 80 mg/day; SMX 400 mg/day	Caspofungin 50 mg/day	70 vs. 52	Mean (SD) 43 (15) vs. 41 (13)	Patients with HIV infection	Mortality	7
Tian, Q., 2021 [14]	Retrospective cohort study	Single-center	January 2017 to December 2019	China	TMP 15–20 mg/kg/day; SMX 75–100 mg/kg/day	Caspofungin 50 mg/day, 70 mg on day 1	135 vs. 143	Overall 34 (19–65)	Patients with HIV infection	Mortality; Positive response rate	8

echinocandin −, TMP–SMX monotherapy; echinocandin +, TMP–SMX + echinocandin combination therapy; HIV, human immunodeficiency virus; SD, standard deviation; SMX sulfamethoxazole; TMP, trimethoprim.

## Data Availability

All data are applicable in the paper.

## Supplementary Materials

The following supporting information can be downloaded at: https://www.mdpi.com/article/10.3390/antibiotics11060719/s1, File S1: PRISMA 2020 Checklist.

## References

[1] Roux A., Canet E., Valade S., Gangneux-Robert F., Hamane S., Lafabrie A., Maubon D., Debourgogne A., Le Gal S., Dalle F. (2014). *Pneumocystis jirovecii* pneumonia in patients with or without AIDS, France. Emerg. Infect. Dis..

[2] Bienvenu A.-L., Traore K., Plekhanova I., Bouchrik M., Bossard C., Picot S. (2016). Pneumocystis pneumonia suspected cases in 604 non-HIV and HIV patients. Int. J. Infect. Dis..

[3] Wickramasekaran R.N., Jewell M.P., Sorvillo F., Kuo T. (2017). The changing trends and profile of pneumocystosis mortality in the United States, 1999–2014. Mycoses.

[4] Mansharamani N.G., Garland R., Delaney D., Koziel H. (2000). Management and outcome patterns for adult *Pneumocystis carinii* pneumonia, 1985 to 1995: Comparison of HIV-associated cases to other immunocompromised states. Chest.

[5] Panel on Opportunistic Infections in HIV-Infected Adults and Adolescents Guidelines for the Prevention and Treatment of Opportunistic Infections in HIV-Infected Adults and Adolescents: Recommendations from the Centers for Disease Control and Prevention, the National Institutes of Health, and the HIV Medicine Association of the Infectious Diseases Society of America. https://aidsinfo.nih.gov/guidelines.

[6] Benfield T., Atzori C., Miller R.F., Helweg-Larsen J. (2008). Second-line salvage treatment of AIDS-associated *Pneumocystis jirovecii* pneumonia: A case series and systematic review. J. Acquir. Immune Defic. Syndr..

[7] Huang L., Crothers K., Atzori C., Benfield T., Miller R., Rabodonirina M., Helweg-Larsen J. (2004). Dihydropteroate synthase gene mutations in Pneumocystis and sulfa resistance. Emerg. Infect. Dis..

[8] Chen P.Y., Yu C.J., Chien J.Y., Hsueh P.R. (2020). Anidulafungin as an alternative treatment for *Pneumocystis jirovecii* pneumonia in patients who cannot tolerate trimethoprim/sulfamethoxazole. Int. J. Antimicrob. Agents.

[9] Beltz K., Kramm C.M., Laws H.J., Schroten H., Wessalowski R., Gobel U. (2006). Combined trimethoprim and caspofungin treatment for severe Pneumocystis jiroveci pneumonia in a five year old boy with acute lymphoblastic leukemia. Klin. Padiatr..

[10] Annaloro C., Della Volpe A., Usardi P., Lambertenghi Deliliers L. (2006). Caspofungin treatment of Pneumocystis pneumonia during conditioning for bone marrow transplantation. Eur. J. Clin. Microbiol. Infect. Dis..

[11] Lu Y.M., Lee Y.T., Chang H.C., Yang H.S., Chang C.Y., Huang C.M., Wei J. (2017). Combination of echinocandins and trimethoprim/sulfamethoxazole for the treatment of pneumocystis jiroveci pneumonia after heart transplantation. Transplant Proc..

[12] Jin F., Liu X.H., Chen W.C., Fan Z.L., Wang H.L. (2019). High initial (1, 3) Beta-d-Glucan concentration may be a predictor of satisfactory response of caspofungin combined with TMP/SMZ for HIV-negative patients with moderate to severe *Pneumocystis jirovecii* pneumonia. Int. J. Infect. Dis..

[13] Wang M., Lang G., Chen Y., Hu C., Guo Y., Tao R., Dong X., Zhu B. (2019). A pilot study of echinocandin combination with trimethoprim/sulfamethoxazole and clindamycin for the treatment of AIDS patients with pneumocystis pneumonia. J. Immunol. Res..

[14] Tian Q., Si J., Jiang F., Xu R., Wei B., Li Q., Jiang Z., Zhao T. (2021). Caspofungin combined with TMP/SMZ as a first-line therapy for moderate-to-severe PCP in patients with human immunodeficiency virus infection. HIV Med..

[15] Kaneshiro E.S., Ellis J.E., Jayasimhulu K., Beach D.H. (1994). Evidence for the presence of “metabolic sterols” in Pneumocystis: Identification and initial characterization of *Pneumocystis carinii* sterols. J. Eukaryot. Microbiol..

[16] Furlong S.T., Samia J.A., Rose R.M., Fishman J.A. (1994). Phytosterols are present in *Pneumocystis carinii*. Antimicrob. Agents Chemother..

[17] Powles M.A., Liberator P., Anderson J., Karkhanis Y., Dropinski J.F., Bouffard F.A., Balkovec J.M., Fujioka H., Aikawa M., McFadden D. (1998). Efficacy of MK991 (L-743,872), a semisynthetic pneumocandin, in murine models of *Pneumocystis carinii*. Antimicrob. Agents Chemother..

[18] Cushion M.T., Collins M.S. (2011). Susceptibility of Pneumocystis to echinocandins in suspension and biofilm cultures. Antimicrob. Agents Chemother..

[19] Lobo M.L., Esteves F., de Sousa B., Cardoso F., Cushion M.T., Antunes F., Matos O. (2013). Therapeutic potential of caspofungin combined with trimethoprim-sulfamethoxazole for pneumocystis pneumonia: A pilot study in mice. PLoS ONE.

[20] Utili R., Durante-Mangoni E., Basilico C., Mattei A., Ragone E., Grossi P. (2007). Efficacy of caspofungin addition to trimethoprim-sulfamethoxazole treatment for severe Pneumocystis pneumonia in solid organ transplant recipients. Transplantation.

[21] Cushion M.T., Linke M.J., Ashbaugh A., Sesterhnn T., Collins M.S., Lynch K., Brubaker R., Walzer P.D. (2010). Echinocandin treatment of pneumocystis pneumonia in rodent models depletes cysts leaving trophic burdens that cannot transmit the infection. PLoS ONE.

[22] Kamboj M., Weinstock D., Sepkowitz K.A. (2006). Progression of Pneumocystis jiroveci pneumonia in patients receiving echinocandin therapy. Clin. Infect. Dis..

[23] Vassallo R., Standing J.E., Limper A.H. (2000). Isolated *Pneumocystis carinii* cell wall glucan provokes lower respiratory tract inflammatory responses. J. Immunol..

[24] Roux A., Gonzalez F., Roux M., Mehrad M., Menotti J., Zahar J.R., Tadros V.X., Azoulay E., Brillet P.Y., Vincent F. (2014). Update on pulmonary *Pneumocystis jirovecii* infection in non-HIV patients. Med. Mal. Infect..

[25] Li W.J., Guo Y.L., Liu T.J., Wang K., Kong J.L. (2015). Diagnosis of pneumocystis pneumonia using serum (1-3)-β-D-Glucan: A bivariate meta-analysis and systematic review. J. Thorac. Dis..

[26] Limper A.H., Offord K.P., Smith T.F., Martin W.J. (1989). Pneumocystis carinii pneumonia. Differences in lung parasite number and inflammation in patients with and without AIDS. Am. Rev. Respir. Dis..

[27] Song J.C., Stevens D.A. (2016). Caspofungin: Pharmacodynamics, pharmacokinetics, clinical uses and treatment outcomes. Crit. Rev. Microbiol..

[28] Nagi A.L., Bourque M.R., Lupinacci R.J., Strohmaier K.M., Kartsonis N.A. (2011). Overview of safety experience with caspofungin in clinical trials conducted over the first 15 year: A brief report. Int. J. Antimicrob. Agents.

[29] The Guidelines of Preferred Reporting Items for Systematic Review and Meta-Analysis (PRISMA) Statement. http://prisma-statement.org.

[30] Moher D., Liberati A., Tetzlaff J., Altman D.G., PRISMA Group (2009). Preferred reporting items for systematic reviews and me-ta-analyses: The PRISMA statement. PLoS Med..

[31] Wells G.A., Shea B., Paterson J. (2000). The Newcastle-Ottawa Scale (NOS) for Assessing the Quality of Nonrandomized Studies in Meta-Analyses. https://www.ohri.ca/Programs/clinical_epidemiology/default.asp.

[32] Kato H., Hagihara M., Asai N., Hirai J., Yamagishi Y., Iwamoto T., Mikamo H. (2022). A systematic review and meta-analysis of efficacy and safety azithromycin versus moxifloxacin for the initial treatment of Mycoplasma genitalium infection. Antibiotics.

